# Not All PET-Avid Endobronchial Lesions Are Malignant: A Case of Chronic Foreign Body Aspiration

**DOI:** 10.3390/reports9020132

**Published:** 2026-04-26

**Authors:** Yordanka Diaz-Saez, Anandu Mathews Anto, Ruchita Kodakandla, Sanjana Voonna, Misbahuddin Khaja

**Affiliations:** Department of Internal Medicine, BronxCare Health System, Bronx, NY 10457, USA

**Keywords:** endobronchial mass, foreign body aspiration, PET positive, vegetable aspiration, post-obstructive pneumonia, middle lobe collapse

## Abstract

Background: Low-dose CT scanning is a key tool in lung cancer screening, enabling the detection of clinically significant abnormalities in asymptomatic individuals and often prompting further diagnostic evaluation. Case Presentation: We describe the case of an 80-year-old man with a heavy smoking history who was found to have a new right middle lobe collapse on screening CT. Subsequent positron emission tomography-computed tomography (PET/CT) imaging demonstrated mild fluorodeoxyglucose (FDG) uptake (SUVmax 2.7), raising concern for a low-grade endobronchial malignancy versus mucoid impaction. Flexible fiberoptic bronchoscopy revealed a large exophytic endobronchial mass occluding the airway. Histopathologic examination of the biopsy sample unexpectedly revealed vegetable material, consistent with chronic foreign-body aspiration. Discussion: Unrecognized aspiration events are relatively common in elderly adults and can mimic malignancy on imaging. This case highlights an important diagnostic pitfall: inflammatory endobronchial processes, including foreign-body granulomas, can demonstrate FDG uptake and mimic malignancy. Conclusion: Clinicians should maintain a broad differential diagnosis when evaluating PET-avid endobronchial lesions, especially in elderly patients.

## 1. Introduction

Aspiration pneumonia accounts for approximately 5–24% of all cases of pneumonia and is particularly prevalent in the elderly population, in whom aspiration events are frequently unrecognized or unreported [1]. The diagnosis of foreign body (FB) aspiration in geriatric patients is often delayed, as many individuals do not recall a choking or aspiration episode; in one study, only 29% of elderly patients reported a clear history of aspiration compared with 50% of younger patients [2]. Commonly aspirated foreign bodies in older adults include food particles such as bone fragments and plant material, components of dental prostheses, and tablets. Chronic or occult FB aspiration may present with nonspecific respiratory symptoms or radiographic findings that mimic malignancy or chronic lung disease, posing a significant diagnostic challenge.

Fluorodeoxyglucose positron emission tomography (FDG-PET) is frequently used to characterize indeterminate pulmonary and endobronchial lesions; however, its specificity is limited. Increased FDG uptake is not exclusive to malignant lesions and may also be observed in inflammatory and infectious conditions, including granulomatous disease, organizing pneumonia, and foreign body reactions. This can lead to false-positive interpretations, particularly in cases of mild-to-moderate uptake.

We report a case of an elderly patient with chronic cough and new lobar atelectasis in whom bronchoscopic evaluation revealed an organic endobronchial foreign body masquerading as a PET-avid endobronchial mass, highlighting the importance of considering clinical, radiological, and bronchoscopic data when evaluating PET-positive lesions to avoid misdiagnosis.

## 2. Case Presentation

**Figure 1:**
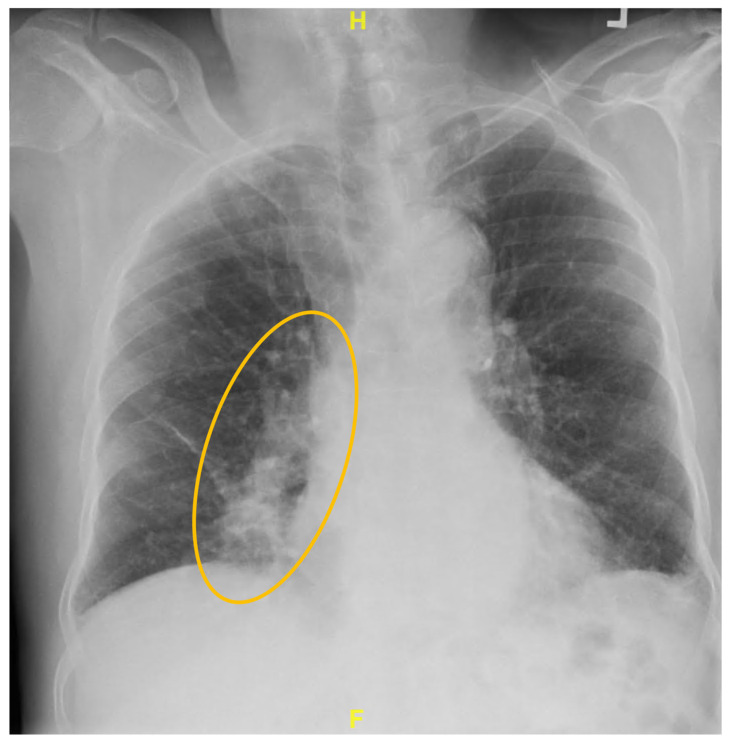
Chest Xray (PA view): Yellow circle demonstrating fibronodular infiltrates in the right middle lobe.

An 80-year-old male patient with COPD emphysema on ICS-LAMA-LABA presented to the pulmonary clinic for COPD management and routine lung cancer screening. The patient reported a chronic dry cough for many years. He is a heavy current smoker with more than a 40 pack-year smoking history. Pulmonary function testing demonstrated an FEV1/FVC ratio of 0.62, which is above the lower limit of normal for age when interpreted using the Global Lung Initiative (GLI-2012) age-adjusted reference equations. The FEV1 was 2.58 L (99% predicted) and the FVC was 4.14 L (119%), with normal lung volumes. The diffusing capacity for carbon monoxide (DLCO) was moderately reduced at 48% predicted (Z-score −3.41). The patient’s physical examination revealed bilateral poor air entry, with mild crackles at the right inframammary and mammary areas and mild wheezing limited to the same region. Routine blood work was within normal limits. X-ray chest revealed fibronodular infiltrates in the right middle lobe (Figure 1).

**Figure 2:**
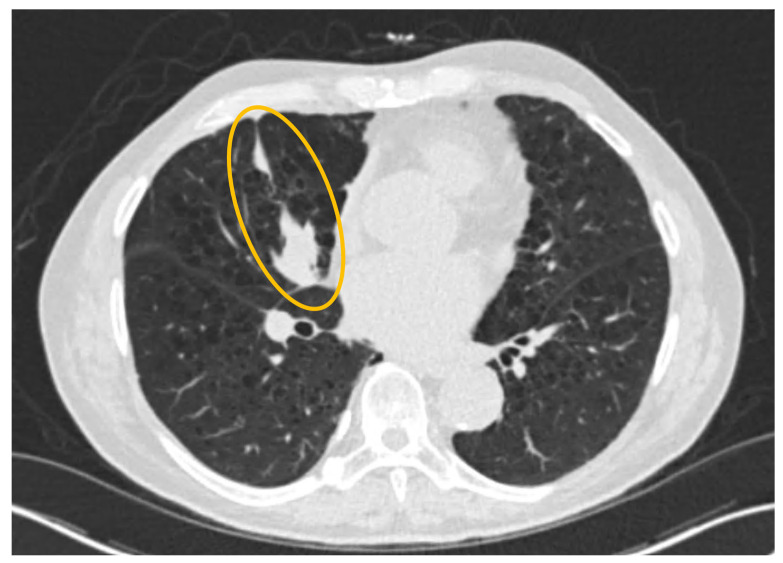
Non-contrast CT chest (lung window): Moderately severe centrilobular emphysema with a yellow circle highlighting the right middle lobe subsegmental atelectasis.

A low-dose CT chest was performed as part of routine lung cancer screening at initial presentation. Low-dose CT of the chest revealed moderately severe centrilobular emphysema with a right middle lobe subsegmental atelectasis. There were no suspicious pulmonary nodules, consolidations, or effusions. A calcified pleural plaque was noted on the right side. No mediastinal masses or lymph nodes were noted, and the tracheobronchial tree appeared to be patent (Figure 2).

He denied any shortness of breath, exertional dyspnea, weight loss, or recent travel and uses one pillow to sleep at night. The patient also denied having any pets.

**Figure 3:**
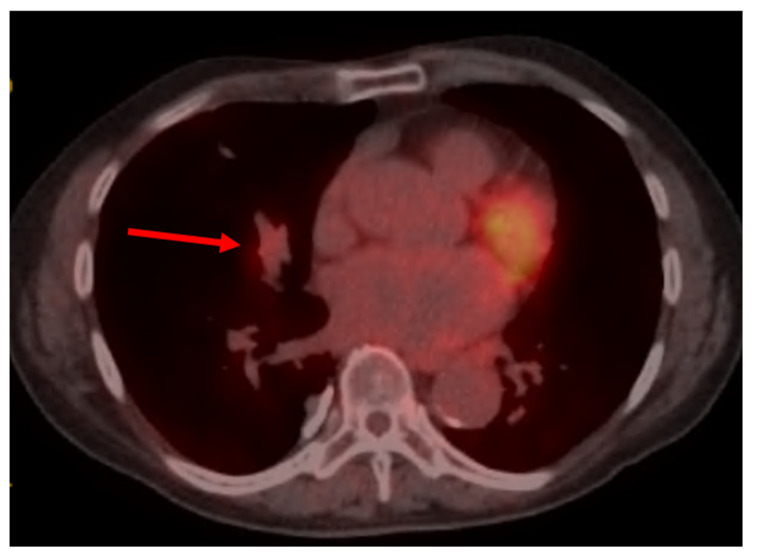
PET/CT scan with a red arrow pointing to the right middle lobe opacity and atelectasis extending from the perihilar region to the periphery of the lung, with mild uptake (SUVmax 2.7) at the perihilar region, suggestive of mucoid impaction versus low-grade endobronchial malignancy.

In view of the right middle lobe subsegmental atelectasis, a PET-CT scan was performed within 2 weeks of the LDCT. PET-CT revealed a linear, branching right middle lobe opacity and atelectasis extending from the perihilar region to the periphery of the lung, with mild uptake (SUVmax 2.7) at the perihilar region, suggestive of mucoid impaction versus low-grade endobronchial malignancy (as reference, the mediastinal blood pool activity demonstrated a maximum SUV of 2.3, and the background activity of the liver demonstrated a maximum SUV of 3.1). PET-CT also highlighted diffuse centrilobular and paraseptal emphysema, biapical scarring, and a few scattered juxtapleural micronodules within both lungs, below PET resolution. A few subcentimeter mediastinal lymph nodes were identified, but they were without increased uptake (Figure 3). The patient was scheduled for an elective flexible bronchoscopy within a week of the PET scan.

**Figure 4:**
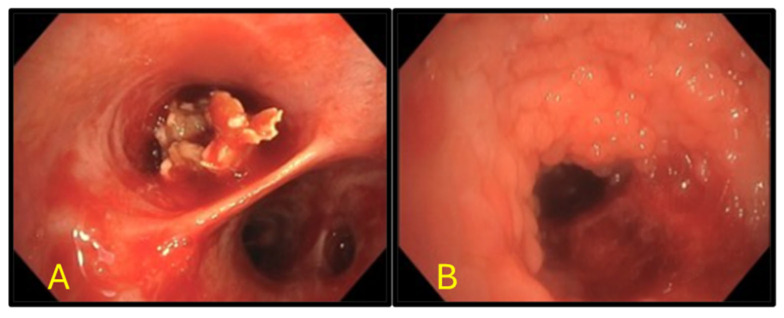
Bronchoscopic images before and after removal of the lesion. (**A**) Right middle lobe exophytic endobronchial lesion occluding >90% of the right middle lobe bronchus. (**B**) Endobronchial lesion removed in toto, with airway mucosa demonstrating a cobblestone appearance in the region of the lesion.

The patient underwent a flexible bronchoscopy using a standard adult flexible bronchoscope (Olympus Corporation, Tokyo, Japan) under moderate sedation. Airway inspection revealed a large, irregular, exophytic, solid mass occluding more than 90% of the right middle lobe bronchus (Figure 4).

**Figure 5:**
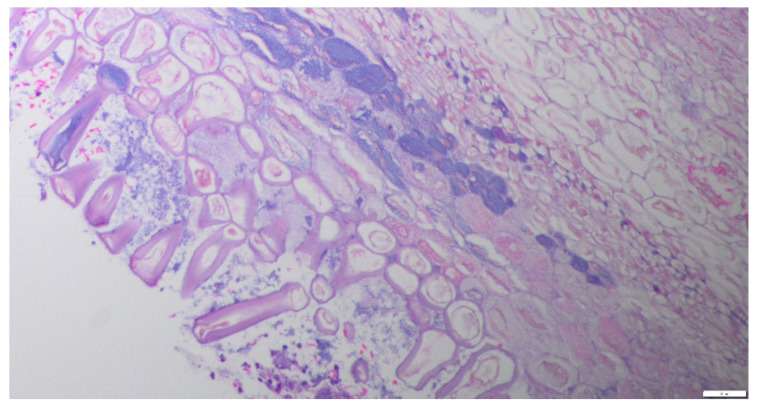
Hematoxylin and eosin–stained section (≈20×) showing vegetable (plant) material characterized by polygonal to elongated cells with thick eosinophilic cell walls and prominent clear vacuoles. The cells lack nuclei and cytologic atypia, producing a pseudo-glandular appearance that may mimic epithelial structures at low power.

Endobronchial brushings and endobronchial biopsy of the mass were obtained. The mass was removed in toto using forceps, restoring airway patency. The surrounding bronchial mucosa in the area showed a cobblestone appearance. The sample was sent for histopathological examination. Bronchoalveolar lavage (BAL) was performed, revealing mucopurulent material, and the sample was sent for cell count, bacterial, fungal, and viral cultures, cytology, AFB, and PJP. BAL cell count revealed neutrophilic inflammation. Cytology and microbiological analysis were negative for malignant cells or bacterial and fungal organisms. Given the thick mucopurulent secretions, the patient was treated with Amoxicillin-Clavulanate for 5 days. Pathology reported fragments of vegetable material—undigested food particles—with mucus and a few inflammatory cells. No fungal elements were seen (Figure 5). The patient was interviewed again, but he denied any history suggestive of aspiration. A focused assessment of aspiration risk was performed. Patient denied dysphagia, gastroesophageal reflux symptoms, alcohol use, or prior choking episodes. He uses dentures but has no trouble chewing or swallowing. No history of neurological or neurodegenerative diseases. Formal dysphagia evaluation was unremarkable. The patient reported improvement in chronic cough following bronchoscopic removal of the foreign body in toto. Given the complete removal of the obstructing lesion and clinical improvement, repeat imaging was not performed immediately. The patient has been scheduled for interval follow-up with repeat imaging in one year as part of routine surveillance.

## 3. Discussion

Our case presented a unique diagnostic challenge. An incidentally detected right middle lobe collapse with a PET-avid endobronchial lesion in an elderly heavy smoker raises significant concern for malignancy. However, this case highlights the importance of maintaining a high degree of suspicion and a broad differential diagnosis, including foreign body aspiration. Over time, an aspirated foreign body can elicit a localized inflammatory response, leading to granulation tissue formation that may mimic a malignant endobronchial mass on imaging.

The PET-CT findings in this case warrant particular attention. The lesion demonstrated mild FDG uptake with a maximum standardized uptake value (SUVmax) of 2.7, which lies between the mediastinal blood pool and liver activity. While higher SUV values are generally associated with malignancy, there is significant overlap between benign and malignant processes [3,4,5]. Inflammatory conditions, including foreign body reactions and granulomatous inflammation, can demonstrate mild to moderate FDG uptake, typically in the range of 2–5 [6]. Many endobronchial malignancies, particularly non-small cell lung cancer, often demonstrate higher SUV values, while low-grade tumors, such as carcinoids, show low FDG avidity [7].

The differential diagnosis of PET-avid endobronchial lesions is therefore broad and includes both malignant and benign conditions. Malignant etiologies include primary lung cancers and carcinoid tumors, while benign conditions that mimic malignancy include foreign body granulomas, endobronchial tuberculosis, sarcoidosis, amyloidosis, and organizing pneumonia [3,4,6]. Recognition of these entities is essential, as reliance on PET imaging alone may lead to misdiagnosis and unnecessary invasive interventions.

Aspiration pneumonia is particularly common among older adults and contributes significantly to morbidity in this population [8]. Identification and removal of the foreign body is pivotal to avoid complications such as recurrent pneumonia, hemoptysis, and granulation formation. Risk factors for aspiration include dysphagia, neurological diseases, impaired functional status, poor oral health, and denture-related swallowing difficulties [9,10,11,12]. Clinical symptoms are either absent or nonspecific, and hence adult airway foreign body aspirations are easily misdiagnosed or delayed by months to years [2,13]. Occult foreign body aspirations have a diagnostic delay due to neglecting the importance of detailing the remote history of FB inhalation, the absence of symptoms during aspiration, and the fact that some patients did not even have a predisposing condition to suggest an aspiration event, and confusion of the sequelae of FB aspiration with bronchiectasis secondary to infections such as TB.

The symptoms suggestive of an aspiration event depend on the site of the foreign body, if the foreign body stays in the trachea, bouts of cough with stridor may be heard; however, if it passes into the bronchi, symptoms can range from minimal to obvious cough, sputum, wheezing, and choking [14]. If clinical symptoms and history are not suggestive of foreign body aspiration, a CT chest may help detect a foreign body. In a study that compares geriatric foreign body aspiration with nongeriatric foreign body aspiration, chest CT diagnosed foreign body in 21% to 35% cases [2]. A flexible bronchoscopy is the next best step, as it can be diagnostic and may even be therapeutic, as in the case of a foreign body. Flexible bronchoscopy is preferred in adults, as it has a greater than 90% success rate for retrieval and allows for airway examination. Great care must be taken to prevent pushing the foreign body distally into the airway [15].

Similar cases have been reported in the literature. Roy et al. described an aspirated almond presenting as an obstructing endobronchial mass suspicious for malignancy. Unlike prior reports, our case is notable for the use of PET-CT, which demonstrated FDG uptake, further increasing suspicion for malignancy. Foreign body aspiration can lead to post-obstructive pneumonia and chronic bronchiectasis. Essential oils found in nuts, such as almonds, can cause irritation, leading to dense inflammatory changes [16]. The reaction of the bronchi and lung parenchyma to a foreign body depends on the type of foreign body. The reaction encompasses acute and organizing inflammation, usually accompanied by one or more of the following: abscess formation, suppurative granulomas, and a giant cell reaction. Aspirated vegetable matter usually incites a giant cell reaction, whereas skeletal muscle fibers usually do not. Vegetable material can be recognized by latticework arrangements of square or rectangular large cells with thick cell walls composed of cellulose. These are found infiltrated with inflammatory cells and surrounded by giant cells. In a histopathological examination of lung tissue, a clue to aspiration in the absence of foreign material and granulomas is the presence of scattered giant cells along with acute and/or organizing pneumonia [17].

An experimental study in a porcine model evaluated the lung’s response to organic vs. inorganic foreign bodies. This study found that lung parenchymal involvement occurred by day 5, whereas shorter exposure durations resulted only in bronchial lesions. Lesion severity did not increase between days 5 and 30, indicating lesion stabilization with limited healing and further supporting the importance of early foreign body removal [18].

There are no consensus guidelines published for the treatment of foreign body aspiration with antibiotics. However, post-obstructive pneumonia, bronchiectasis, and lung abscess are reported complications and may warrant antibiotic coverage. In view of the thick mucopurulent secretions we observed during bronchoscopy, we gave the patient 5 days of antibiotic coverage, following which his symptoms (chronic cough) improved [19]. Possibly the foreign body was aspirated months ago, and the patient did not notice the aspiration event, leading to a foreign body reaction around the vegetable foreign body in the middle lobe of the right lung, which was kept walled off, with surrounding bronchi showing a cobblestone reaction to the foreign body.

## 4. Conclusions

This case highlights an important limitation of FDG-PET imaging, as mild to moderate FDG uptake does not reliably distinguish malignant from inflammatory endobronchial processes. Foreign body aspiration should be considered in the differential diagnosis of PET-avid endobronchial lesions, particularly in elderly patients, even in the absence of a clear aspiration history. Bronchoscopy remains essential for definitive diagnosis and management.

## Data Availability

The original data presented in the study are included in the article; further inquiries can be directed to the corresponding author.

## Abbreviation

AFB	Acid-fast bacilli
BAL	Bronchoalveolar lavage
COPD	Chronic obstructive pulmonary disease
CT	Computed tomography
DLCO	Diffusing capacity of the lung for carbon monoxide
FB	Foreign body
FEV1	Forced expiratory volume in one second
FVC	Forced vital capacity
ICS	Inhaled corticosteroid
LABA	Long-acting beta-agonist
LAMA	Long-acting muscarinic antagonist
PET	Positron emission tomography
PET-CT	Positron emission tomography–computed tomography
PJP	Pneumocystis jirovecii pneumonia
RML	Right middle lobe
SUV	Standardized uptake value
TB	Tuberculosis
